# Targeted and Untargeted Metabolic Profiling of Wild Grassland Plants identifies Antibiotic and Anthelmintic Compounds Targeting Pathogen Physiology, Metabolism and Reproduction

**DOI:** 10.1038/s41598-018-20091-z

**Published:** 2018-01-26

**Authors:** Katherine E. French, Joe Harvey, James S. O. McCullagh

**Affiliations:** 1 Department of Plant Sciences, University of Oxford, South Parks Road, Oxford, OX1 3RB UK; 2 Department of Chemistry, University of Oxford, Mansfield Road, Oxford, OX1 3TA UK

## Abstract

Plants traditionally used by farmers to manage livestock ailments could reduce reliance on synthetic antibiotics and anthelmintics but in many cases their chemical composition is unknown. As a case study, we analyzed the metabolite profiles of 17 plant species and 45 biomass samples from agricultural grasslands in England using targeted and untargeted metabolite profiling by liquid-chromatography mass spectrometry. We identified a range of plant secondary metabolites, including 32 compounds with known antimicrobial/anthelmintic properties which varied considerably across the different plant samples. These compounds have been shown previously to target multiple aspects of pathogen physiology and metabolism *in vitro* and *in vivo*, including inhibition of quorum sensing in bacteria and egg viability in nematodes. The most abundant bioactive compounds were benzoic acid, myricetin, p-coumaric acid, rhamnetin, and rosmarinic acid. Four wild plants (*Filipendula ulmaria* (L.) Maxim.*, Prunella vulgaris* L., *Centuarea nigra* L., and *Rhinanthus minor* L.) and two forage legumes (*Medicago sativa* L., *Trifolium hybridium* L.) contained high levels of these compounds. Forage samples from native high-diversity grasslands had a greater abundance of medicinal compounds than samples from agriculturally improved grasslands. Incorporating plants with antibiotic/anthelmintic compounds into livestock feeds may reduce global drug-resistance and preserve the efficacy of last-resort drugs.

## Introduction

Plants are known to produce over 60,000 secondary metabolites to protect themselves from microbial pathogens and herbivores^1^. These chemicals could be exploited to reduce antimicrobial and anthelmintic drug use in livestock, and in the future, in humans. Growing livestock antibiotic and anthelmintic resistance is a major concern among farmers worldwide^2,3^. For example, globally, over 50,000 tons of antibiotics are given to livestock per year yet many are now ineffective^4,5^. Resistance to anthelmintics in sheep can reduce livestock productivity by up to 50% and increase the mortality rate of calves and lambs^6,7^. Intensified livestock production, misuse of antibiotic and anthelmintics, and changes in livestock diet, have all contributed to this process. Resistant pathogens in livestock can also infect humans through direct contact with sick animals and agricultural run-off contaminating arable fields and waterways^8,9^. In the EU alone, the annual economic cost of antimicrobial resistance in humans is estimated at over £1.5 billion (due to healthcare expenses and lost productivity) and causes over 25,000 deaths annually^10^. Global and national antimicrobial strategies call for the development of novel treatments and new drugs to reduce antimicrobial resistance but few have looked at plant compounds as a solution^11^. Current research focuses on identifying new antimicrobial and/or anti-parasitic properties from new or rare microbiota or developing lysins, probiotics, and genetically-engineered phages to deactivate specific microbial defense mechanisms^12–14^. However, these are temporary solutions and will not overcome resistance in the long term. Microbial and parasitic pathogens continually develop resistance to compounds from other bacteria and fungi and rapidly evolve defense mechanisms against treatments targeting specific genetic sequences and surface proteins.

Grassland plants in particular may be a rich and untapped source of bioactive compounds^15–17^. Traditionally, plants from the Asteraceae and Lamiaceae families, such as *Artemesia vulgaris, Nepeta cataria, and Mentha* spp., were associated with antibacterial and de-worming properties. However, the chemical composition of many wild grassland plants and the biosynthesis of the antimicrobial and anthelmintic compounds they contain is unknown. In addition, to date it is uncertain whether adding a few species with medicinal compounds into agricultural grasslands can affect the overall chemical composition of the entire sward.

Here, we use liquid-chromatography mass spectrometry and metabolic network analysis to investigate variability in the antimicrobial and anthelmintic compounds found in 17 grassland plant species in Oxfordshire, England. We examined whether known antimicrobial and anthelmintic compounds were species-specific or shared across genera and we identified which plants contained elevated levels of these compounds. We also examined the availability of antimicrobial and anthelmintic compounds in 45 mixed-forage samples from current agricultural grasslands to determine how species-composition affects the overall chemical composition of grassland biomass.

## Methods

### Plant collection

We collected plant samples from nine sites in June 2016. This included 17 common grassland species: *Achillea millefolium* L.*, Centaurea nigra* L.*, Cichorium intybus* L., *Filipendula ulmaria* (L.) Maxim.*, Lathyrus pratensis* L., *Leucanthemum vulgare* Lam., *Lotus corniculatus* L., *Medicago lupulinum* L., *Medicago sativa* L., *Onobrychis viciifolia* Scop., *Plantago lanceolata* L., *Prunella vulgaris* L.*, Rhinanthus minor* L.*, Stachys sylvatica* L.*, Trifolium hybridum* L., *Trifolium pratense* L., *Vicia cracca* L., and *Vicia sativa* L. All of these are wild plants except for *Medicago sativa, Onobrychis viciifolia*, *Trifolium hybridum*, and *Cichorium intybus*. Plant sampling and preparation for metabolite extraction were performed according to previously established guidelines^17–21^. For each of the 17 species, four plants were collected. Individual plants were at least 1 m apart. Plants were cut 5 cm from the ground and collected at roughly the same time of day (10–11 am). Only plants free of visible microbial infection were collected^22^. The individual plants were freeze dried for 48 hours upon collection. We also collected forage sample of ca. 200 grams from nine grassland sites. These grasslands were divided into three experimental groups: semi-natural, species-rich grasslands; herbal leys; and improved grasslands. Semi-natural grasslands are high in native biodiversity and have never been agriculturally improved. Herbal leys are short-term leys sown with plants specifically thought to promote livestock health, primarily lucerne (*Medicago sativa*), sainfoin (*Onobrychis viciifolia*) and chicory (*Cichorium intybus*). Improved grasslands are sown annually or biannually with 1–3 grass species (ryegrass (*Lolium perenne*), festuliolium (*Festuca* x *Lolium*), and smooth Fescue (*Festuca pratensis*)). To obtain representative samples from each site we created five 1 m^2^ plots 5 m apart and collected 20 grass samples from each plot. Small handfuls of herbage were cut 5 cm above the soil. Each sample consisted of all the plants in each handful; no plants were removed. Samples were collected within the same time period (10–11 am) to ensure comparability among samples. The mixed forage samples were dried on newspaper for one week in a dark seed-drying room to remove excess water.

### Metabolite extraction

We homogenized each sample using a food-processor, suitably washed between samples to avoid cross-contamination. 600 mg of each sample was placed in 15 mL Precellys homogenizer tubes with large ceramic (zirconium dioxide) beads and homogenized for 7 minutes using a Precellys Evolution homogenizer (Bertin Instrument, Montigny-le-Bretonneux, France). 5 mL of 80% ethanol was added to each sample and homogenized again for 7 minutes. The ceramic beads were removed and the mixture was poured into 15 mL Falcon tubes. The homogenizer tubes were washed with an additional 3 mL of 80% ethanol which was added to the mixtures in the Falcon tubes. Samples were centrifuged at 3260 g at 25° Celsius for 23 minutes. 1 mL of each sample was filtered using 0.45 µm ptfe filters into a standard autosampler vial and then samples were stored at −80 °C until the day of analysis. During storage, a precipitate formed in a small number of samples. These were specific to several legume species. It was presumed (but not proven) that this was due to protein or tannic precipitation as a result of the relatively high tannin content (also previously reported)^23^. All samples were re-filtered using a 0.45 µm filter prior to analysis to remove any residual precipitate. We confirmed that the filtration process did not affect the relative abundance and diversity of metabolites detected (Supplementary Dataset 1; Supplementary Figure 1).

### LC-MS analysis

Samples were analyzed using targeted and untargeted metabolomics profiling using liquid-chromatography-tandem mass spectrometry (LC-MS/MS). The method used was based on a modified version of that published by Want and co-workers^24^. A Thermo Scientific Ultimate 3000 liquid-chromatography system was coupled directly to a Q-Exactive HF Hybrid Quadrupole-Orbitrap mass spectrometer with a HESI II electrospray ionisation source (Thermo Scientific, San Jose, CA). A 10 μL partial loop injection was used for all analyses and the chromatographic separation was performed using a Waters Acquity UPLC HSST3 column with dimensions 2.1 × 100 mm and 1.7 uM particle size. The flow rate was 0.3 mL/min. The total run time was 12 minutes and the mobile phase composition was as follows: mobile phase A 0.1% aqueous formic acid and mobile phase B HPLC grade MeOH in 0.1% formic acid. A linear gradient was used as follows: 0 minutes, 1% B; 1 minute, 1% B; 8 minutes, 95% B; 9 minutes, 95% B; 9.1 minutes, 1% B; 12 minutes, 1% B. Analysis was performed in negative ion mode using a scan range from *m/z* 60–900 Da and resolution set to 70,000. The tune file source parameters were set as follows: Sheath gas flow 60; Aux gas flow 20; Spray voltage 3.6 kV; Capillary temperature 320 °C; S-lens RF value 70; Heater temperature 300 °C. AGC target was set to 1e^6^ and the Max IT value was 250 ms. The column temperature was kept at 30 °C throughout the experiment. Peaks retention times were identified from the injection of authentic standards at 5 µg/mL concentration in milli-Q water and metabolite identification was performed using a combination of accurate mass analysis (<5 ppm), retention time <0.5 min, MS/MS peak matching and isotope pattern matching. Data processing and identification of metabolites was made using Thermo Scientific Xcaliber (Thermofisher Scientific, Hemmel, UK) and Waters Progenesis QI (Waters, Elstree, UK).

### Method validation

#### Limit of detection (LOD) and limit of quantification (LOQ)

Relative abundances for all measured compounds were determined by integration of the peak area from their extracted ion chromatograms and compared across samples. For the identified compounds where authentic standards were available, the LOD for each metabolite was determined based on a serial dilution of an authentic standard until a signal to noise ratio of 3:1 compared to baseline noise was reached. The LODs were in the range 4.2 pg/mL to 66.7 ng/ml. No blank matrix was available for these experiments so all authentic standards were added into one sampling vial at a concentration of 1 mg/mL. From this solution, ten serial dilutions were created ranging from 5 µg/mL to 2.56 pg/mL.

LOQs were determined by creating a separate calibration curve for each compound and identifying the lowest concentration in the linear range of the curve which had a signal: noise >10:1. The limits of quantification were in the range 12.8 pg/mL and 200 ng/mL for the identified compounds for which authentic standards were available. A table of LOD and LOQ values for each compound measured is presented in Supplementary Table 1. Supplementary Figure 2 shows an example of one of these calibration curves for trans-ferulic acid. The R^2^ values for all curves were >0.99 (Supplementary Table 1).

#### Precision

Analytical precision was determined by eight replicate measurements. The result was expressed as a relative standard deviation (%RSD). The results for quercetin are shown as an example in Supplementary Table 2.

#### Compound Concentrations

The calibration curves created for each compound were used to estimate their concentration in each sample. The equation of the line for each calibration curve was used to calculate the concentration from the peak area for that compound in each sample. All concentrations were given in ng/ml (Supplementary Dataset 2).

### Identification of plant metabolites and data analysis

Plant metabolites were identified principally using values obtained from the analysis of authentic standards. We measured 110 plant secondary compounds and determined chromatographic retention times, accurate monoisotopic mass values, natural abundance isotope patterns and HCD fragmentation patterns. To ensure accurate identification of compounds in the plant samples we matched these four independent criteria for each compound with those of the authentic standards. Compound identifications where only accepted when the following criteria were met: a m/z error of less than 5 ppm when compared to the theoretical value, a chromatographic retention time <20 seconds of the authentic standard (ran separately), an isotope similarity match >95% compared to the theoretical value based on the chemical formula. Where recorded using a data dependent fragmentation method we also compared fragmentation patterns to confirm compounds identifications (at least base peak and two additional fragments). Finally, we used the Human Metabolome Database (HMDB) database (www.hmdb.ca), containing over 18,000 compounds found in the human body (many of which are from plants consumed as part of the human diet), to assign *putative identifications* to compounds based on mass error and isotope similarity and theoretical fragmentation pattern matching (where possible)^25^. We clearly identify in the results where compound were identified in this way and not by direct matching to authentic standards. Processing of mass spectra and identification of compounds was conducted using Progenesis QI (v.2.2) software. To identify and visualize the biosynthetic pathways associated with each compound, we used the OmicsViewer developed by the Plant Metabolic Network (PMN)^26^.

### Statistical analysis

All data was log-transformed and normalized before statistical analysis. To determine the overall metabolomic variation in individual species and grassland types, we used Principal Component Analysis (PCA) and partial least squares–discriminant analysis (PLS-DA). Because PLSDA can over fit data, we used 1000 permutations to validate these models^27^. To visualize the relationship among samples and among metabolites, we used hierarchal clustering with Euclidean distances as the similarity measure and Ward’s linkage as the clustering algorithm^28^. Results were visualized in the form of dendrograms and heat maps. We conducted one-way ANOVAs followed by a post-hoc analysis using the Tukey HSD test to determine which compounds varied significantly among the samples. To determine whether the concentration of specific compounds was higher in specific plants (as identified in the heat maps) was statistically significant, we used multiple comparisons of means tests. PCA, PLS-DA, dendrograms and heat maps were created using MetaboAnalyst 3.0^28^. General statistics, ANOVA, and multiple comparison of means tests were conducted in R (v. 3.2.2, “Fire Safety”) using packages *stats* (v. 3.4, R core team), *psych* (v. 1.6.4), and *multcomp* (1.4.6)^29,30^.

## Results

### Untargeted Metabolic analysis of single plant species

We measured 22,293 ion features in total across all 68 samples using untargeted analysis LC/MS analysis. This translated into 16,260 compounds measured with a CV < 30 and an abundance > 500 using automated data processing software (Progenesis QI). From our in-house plant secondary metabolite database containing 110 authenticated standards (Supplementary Table 3), 51 identifications were confirmed of which 26 have previously been shown to have antimicrobial and/or anthelmintic properties. A further 113 compounds were putatively identified based on accurate mass, isotope pattern and theoretical fragmentation pattern matching criteria using the HMDB structural database. Next we quantified the identified compounds for which we had authentic standards using an external calibration approach. The most abundant compounds, with confirmed identifications and calculated concentrations, were benzoic acid (μ = 70.9 ± 21.7 ng/g), mannitol (μ = 55.1 ± 16.9 ng/g), and chicoric acid (μ = 40.3 ± 11.5 ng/g) while the least abundant were hesperetin (μ = 0.01 ± 0.001 ng/g), chrysin (μ = 0.001 ± 0.0001 ng/g), and luteolin-7-O-glucuronide (μ = 0.008 ± 5.6E-05 ng/g) (Supplementary Dataset 2).

**Figure 1:**
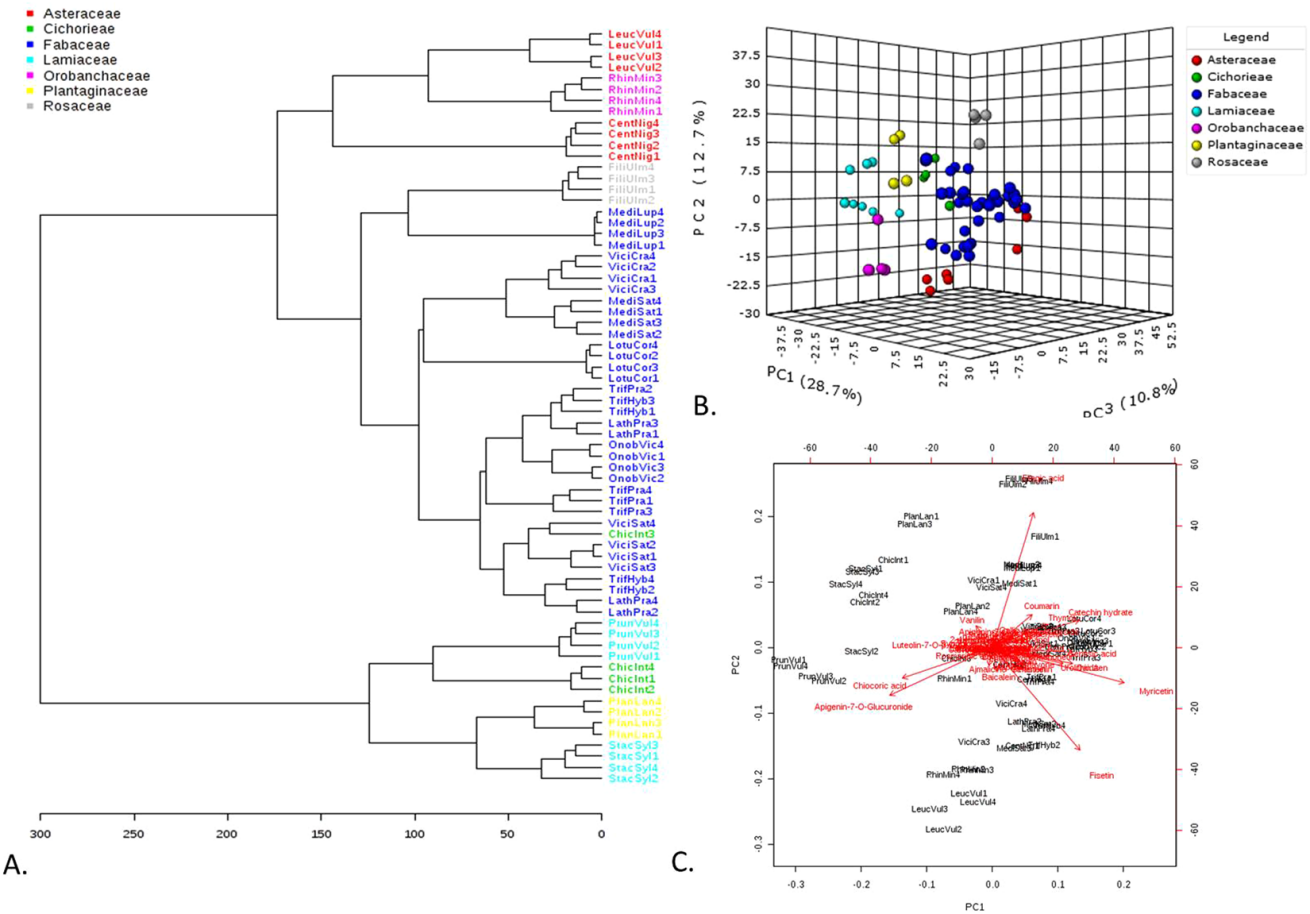
Phytochemical composition has a phylogenetic basis. (**A**) Phylogenetic relationships among the samples are highlighted in the chemotaxonomy of 17 grassland plants based on Euclidean distances and Ward clustering. (**B**) PCA of metabolomic composition of different grassland plant species showing components 1, 2 and 3. (**C**) Biplot of the PCA in Fig. 1A showing the compounds causing the strongest divergence among the samples. In all figures, samples from each plant are identified as the first four letters of the genus and first three letters of the species according to Linnean classification. Samples are color coded by Family. See Supplementary Figure 4 for PCA and PLDA graphs color coded by species instead of family.

PCA and PLS-DA analyses of all measured compounds (Fig. 1) showed divergence between samples based in part on phylogeny. Samples of plants from the Fabaceae family formed a tight group (Fig. 1A). Clustering was also seen for other families where more than one species was represented, which can be seen more clearly in the 3-D PCA scores plot and in PLS-DA analysis (Fig. 1B). The biplot for PC1 and PC2 showed that specific chemicals were associated with specific families: Asteraceae (fisetin), Fabaceae (coumarin, thymol, catechin, myricetin), Rosaceae and Plantagenaceae (fumic acid), and Lamiaceae (chicoric acid, apigenin-7-*O*-glucorinide) (Fig. 1C). The dendrograms created from this data also showed the samples dividing according to phylogeny, and specifically, into two general groups: Plantaginaceae-Cichorieae-Lamiaceae and Fabaceae-Rosaceae-Asteraceae-Orobanchaceae (Supplementary Figure 2). These closely associated families are related at the level of order or clade. The one exception is Cichorieae, which according to phylogeny should have grouped more closely with plants from the Asteraceae family (they are from the same order, Asterales). These data show that genetic diversity is correlated with chemical diversity at least for the selected plants analyzed.

**Figure 2:**
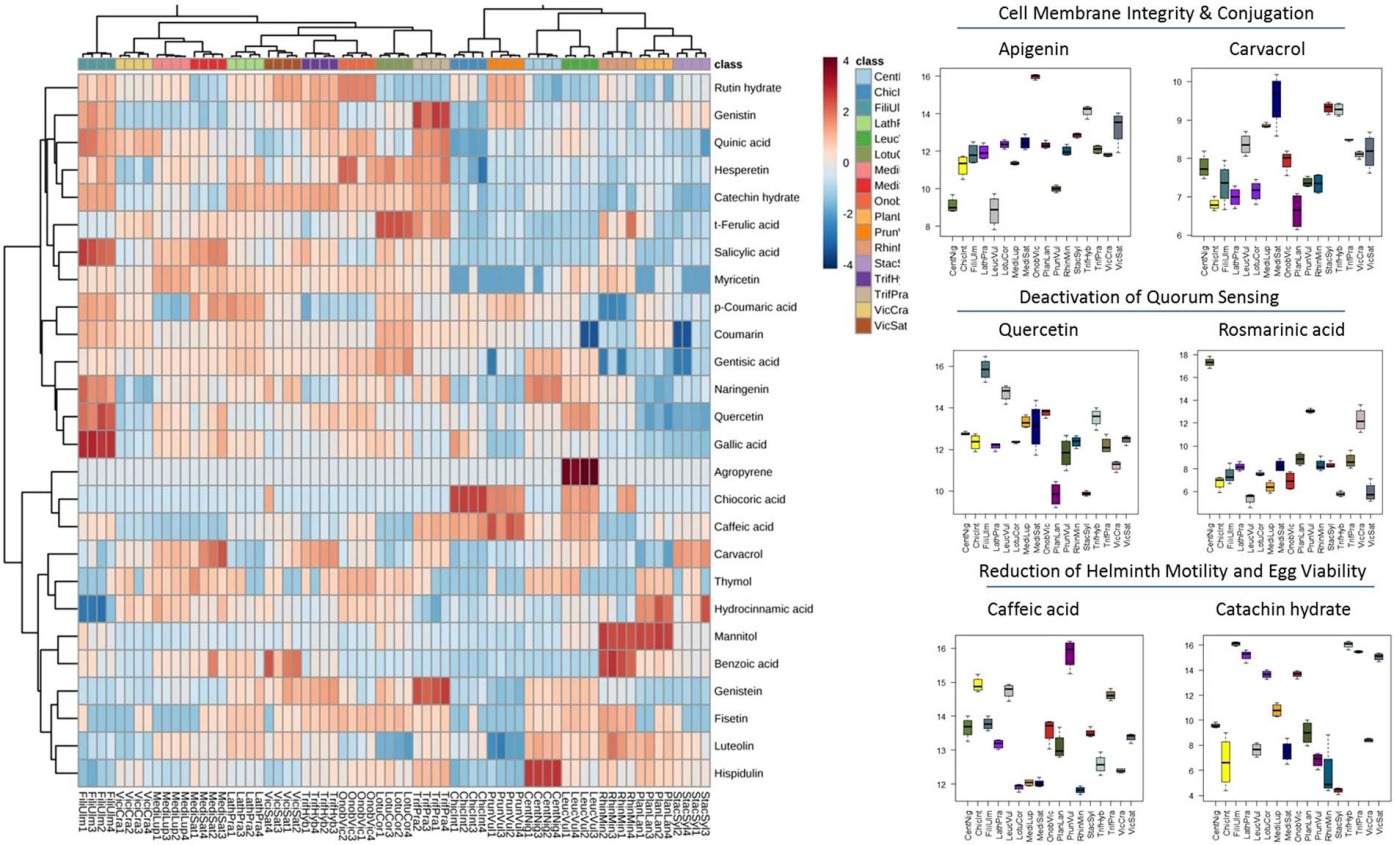
Heat map of 31 antimicrobial and anthelmintic compounds found across 17 grassland plants. These compounds perform a diverse array of functions, including targeting microbial cell membrane integrity and conjugation in bacteria, disrupting quorum sensing, and reducing the motility and fertility of intestinal worms. Samples from each plant are identified as the first four letters of the genus and first three letters of the species according to Linnean classification. Compound concentrations are represented on a log scale.

In general, compounds with previously identified antimicrobial and/or anthelmintic properties were found across all the species analyzed but at significantly different concentrations. The mean concentration of all compounds varied significantly between the different species, the most significant of which were syringic acid (*F*_16,51_ = 204.46, p < 0.001), formononetin (*F*_16,51_ = 159.08, p < 0.001), vanilin (*F*_16,51_ = 138.08, p < 0.001), and mannitol (*F*_16,51_ = 122.02, p < 0.001). Figure 2 presents a heat map showing the relative concentration of all antimicrobial and anthelmintic compounds in each sample (a heat map for all compounds present in the samples is in Supplementary Figure 5). Many of the most abundant metabolites were shared among all the species analyzed. However, certain plants, especially *Filipendula ulmaria*, *Centurea nigra*, *Prunella vulgaris*, and *Rhinanthus minor*, contained elevated levels of specific compounds (Supplementary Figure 6). For example, *Filipendula ulmaria* contained on average higher levels of salicylic acid than any other species analyzed in the study (15.85 ± 0.15, *t* = 21.4, p < 0.001). A few compounds were species specific. Agropyrene was only found in *Leucanthemum vulgare*. Benzoic acid was found primarily in *Rhinanthus minor* (15.29 ± 0.31) with much lower levels in two other plants (*Vicia sativa* and *Medicago sativa*). A similar trend was observed for chicoric acid, which was highly abundant in *Cichorium intybus* (11.45 ± 0.08) and at very low levels in *Leucanthemum vulgare* (5.24 ± 0.32) and *Prunella vulgaris* (6.58 ± 0.31).

There were also differences in the bioactive compounds found in closely related plants. Our study contained two forage crops (*Medicago sativa*, *Vicia sativa*) and their wild relatives (*Medicago lupulina*, *Vicia cracca*). There was no indication that wild or cultivated plants contained significantly different amounts of antimicrobial or anthelmintic compounds. For example, luteolin was more abundant in *Vicia sativa* (14.25 ± 0.23) than its wild relative *Vicia cracca* (12.49 ± 0.17) (*t* = −6.868, p < 0.001). In contrast, quinic acid was more abundant in *Vicia cracca* (11.51 ± 0.05) than *Vicia sativa* (10.69 ± 0.07) (*t* = −7.728, p < 0.001).

The anthelmintic compounds we identified in grassland plants were mainly essential oils and tannins. Carvacrol was highly abundant in *Medicago sativa* (9.54 ± 0.35), *Stachys sylvatica* (9.32 ± 0.07), *Trifolium hybridum* (9.27 ± 0.08) and *Medicago lupulina* (8.86 ± 0.03). Thymol was most abundant in *Onobrychis viciifolia* (6.56 ± 0.12), *Medicago sativa* (5.58 ± 1.1) and *Plantago lanceolata* (5.24 ± 0.21). High levels of mannitol was found in *Plantago lanceolata* (16.72 ± 0.05) and *Rhinanthus minor* (16.65 ± 0.08). Catechin was abundant in *Filipendula ulmaria* (16.1 ± 0.08), *Trifolium hybridum* (16.02 ± 0.04), *Trifolium pratensis* (15.46 ± 0.04), and *Lathyrus pratesis* (15.18 ± 0.22). Caffeic acid was significantly more abundant in *Prunella vulgaris* than any other plant (15.84 ± 0.02, *t* = 21.98, p < 0.001).

Of the putative identifications, nine were tannins and saponins with anthelmintic properties. Epicatechin-(6′- > 8)-epicatechin, procyanidin C1 and medicoside J were highly abundant in legumes and *Filipendula ulmaria*. Trygopogonsaponin was found in legumes and *Rhinanthus minor*. Goyasaponin I was found in *Lathyrus pratensis* and *Centaurea nigra*. *Prunella vulgaris* and *Plantago lanceolata* contained saponin H. Chienoside II was only found in *Trifolium hybridum* and assamsaponin E and D were only found in *Prunella vulgaris*.

Currently, the biosynthetic pathways of 78% (27/31) of the compounds identified in the 17 grassland plants samples we sampled have been elucidated in the literature (Supplementary Table 4). The bioactive compounds identified in this study were produced by 22 secondary metabolic pathways. The remaining compounds were produced by eight pathways involved in fatty acid biosynthesis, cell structure, amine/polyamine biosynthesis, carbohydrate biosynthesis, and aromatic compound degradation. Correlation analysis revealed three clusters of highly correlated compounds (Table 1; Supplementary Figures 7 and 8).

**Table 1:** Highly correlated compounds. Hierarchal clustering using Pearson correlation divided metabolites into three groups of highly correlated compounds.

	Compounds	Biosynthetic pathways
Group 1	gentisic acid, urolithin A, narigenin, kaempferol, rhamnetin, fisetin, myricetin, genistein, erodictol, arachidic acid, hispidulin, chrysin, carvacrol, thymol, emodin, lauric acid, amentoflavone	1. syringetin biosynthesis, 2. monoterpene biosynthesis, 3. myricetin gentiobioside synthesis, 4. palmirate biosynthesis*, 5. ω-hydroxylation of coprate and laurate*, 6. ω -hydroxylation of laurate*
Group 2	gentisin, salicylic acid, quinic acid, catechin, daidzin, quercetin, coumarin, trans-ferulic acid, hesperetin, rutin hydrate, formononetin, gallic acid, homovanilic acid, p-coumaric acid, ellagic acid	1. phenylopropanoid biosynthesis**, 2. suberin monomers biosynthesis**, 3. gallate biosynthesis***, 4. salicilate biosynthesis, 5. coumarin biosyntheiss, 6. superpathway of scopulin and esculin biosynthesis, 7. flavonoid biosynthesis, 8. salidroside biosynthesis, 9. quercetin glycoside biosynthesis, 10. free phenylopropanoid biosynthesis, 11. umbelliferone biosythesis, 12. ferulate and sharpate biosyntheis, 13. volatile benzoid biosynthesis, 14. flavonol biosynthesis
Group 3	vanillin, apigenin, syric acid, caffeic acid, chicoric acid, hydrocinnamic acid, styrene, benzoic acid, mannitol, luteolin, rosmarinic acid, baicalein, ellaidic acid, ajmalicine	1. benzoyl-B-D-glucopyranose biosynthesis, 2. simple coumarin biosynthesis, 3. chrysoeriol biosynthesis, 4. luteolin glycosides biosynthesis, 5. benzoate biosynthesis, 6. rosmarinic acid biosynthesis, 7. flavonoid biosynthesis, 8. volatile benzenoid biosynthesis^**†**^, 9. mannitol biosynthesis, 10. benzoate degradation^**‡**^

The biosynthetic pathways covered by each group were identified using the Plant Metabolic Network Pathway Tool. A heat map showing the correlations among compounds is in Supplementary Materials Figure 7. All compounds are part of secondary metabolic networks unless otherwise noted. Compounds formed in other biosynthetic pathways are denoted as follows: *fatty acid biosynthesis; **cell structure; ***amine/polyamine biosynthesis; ^†^carbohydrate biosynthesis; ^‡^aromatic compound degradation.

### Untargeted Metabolic profiling of mixed-plant samples

There were 20,798 compounds measured in total across all 45 samples. Of these, 48 had confirmed identifications of which 32 had antimicrobial and/or anthelmintic properties. A further 118 compounds were putatively identified. Of the confirmed identifications, the most abundant compounds were chicoric acid (μ = 51.6 ± 14 ng/g), mannitol (μ = 39.4 ± 16.3 ng/g), and chlorogenic acid (μ = 13.8 ± 1.4 ng/g) while the least abundant were luteolin-7-O-glucuronide (μ = 0.013 ± 0.004 ng/g), hesperetin (μ = 0.008 ± 0.002 ng/g), and chrysin (μ = 0.001 ± 2.53E-05 ng/g) (Supplementary Dataset 2).

**Figure 3:**
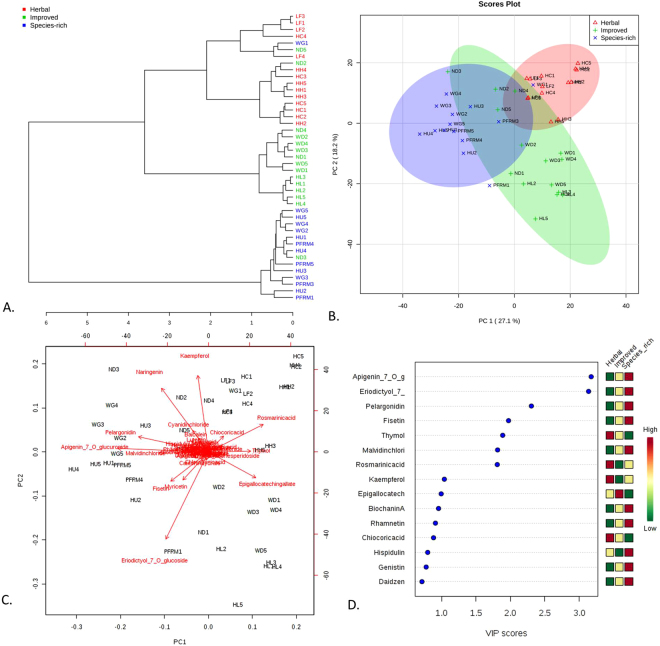
Forage phytochemical composition is influenced by grassland type. (**A**) Dendrogram showing the relationship among the samples using Euclidean distances and Ward clustering. (**B**) PCA plot showing the spatial division among grassland types. (**C**) Biplot of the PCA plot in Fig. 3B showing the compounds most responsible for the divergence among grassland types. (**D**) Key compounds separating the grassland types based on variable importance in projection (VIP) in PLS-DA analysis.

The dendrogram in Fig. 3A shows that the mixed forage samples divide into roughly three groups. The herbal leys, as a group, were slightly more heterogenous than the other groups: samples from one species-rich grassland (WG1) and two improved grasslands (ND2 and ND5) were closely similar to the metabolomic composition of samples from the herbal leys. One sample from an ‘improved grassland’ (ND3) was closely related in metabolomic composition to the samples from the species-rich grasslands. The samples also divided into roughly three groups in principal component analysis (Fig. 3B,C). Samples ND2, ND3, and ND5 (from one ‘improved grassland’) fell in between samples from the species-rich grasslands and herbal leys while WG1 can clearly be seen among the samples form the herbal leys. The bi-plot of the principal components 1 and 2 explains 45.3% of the variation in the data set and indicates several compounds associated with each of the three experimental groups were responsible for the divergence among the samples. Narigenin, fisetin, myricetin, and eriodictyol-7-O-glucoside were associated with species-rich grasslands; kaempferol, rosmarinic acid, chicoric acid, and malvidin chloride were associated with herbal leys; and epigallocatchin gallate was associated with improved grasslands. PLS-DA analysis confirmed the division among the three grassland types; the model was highly significant (p < 0.001) after 1000 permutations. Figure 3D shows the compounds with the greatest impact on the division among the samples.

**Figure 4:**
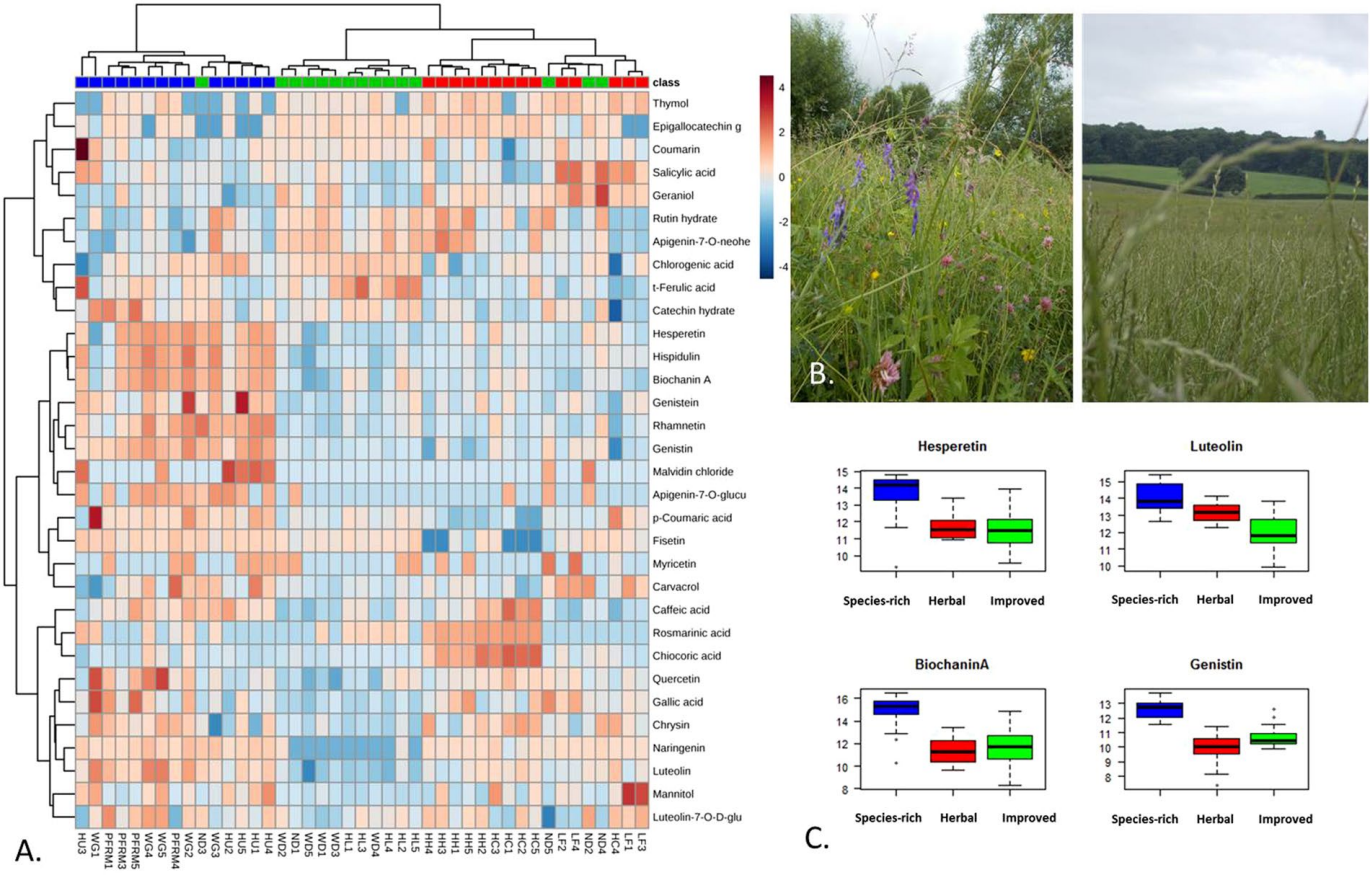
Species-rich grasslands are a hotspot for antimicrobial compounds. (**A**) Heat map of the 31 antimicrobial and anthelmintic compounds found in the mixed forage samples based on Euclidean distances and Ward clustering. (**B**) Comparison of vegetation characteristic of species-rich grasslands (left) and improved grasslands (right). (**C**) Example compounds significantly more abundant in species-rich grasslands. Compound concentrations are represented on a log scale.

The heat map in Fig. 4 indicates antimicrobial and anthelmintic compounds were found primarily in the samples from species-rich grasslands and secondarily in herbal leys. Eight antimicrobial compounds were significantly more abundant in species-rich grasslands compared to the other grassland types. These compounds were biochaninin A (14.72 ± 0.44; *t = *6.03, p < 0.001), luteolin (14.09 ± 0.23; *t* = 5.43, p < 0.001), hispidulin (13.14 ± 0.47; *t* = 6.73, p < 0.001), diadzin (13.17 ± 0.25; *t* = 7.12, p < 0.001), hesperetin (13 ± 0.32; *t* = 5.012, p < 0.001), genistin (12.65 ± 0.24; *t = *7.86, p < 0.001), genistein (12.56 ± 0.21; *t* = 5.079, p < 0.001), and rhamnetin (11.23 ± 0.38; *t* = 6.30, p < 0.001). In a few cases, specific compounds like caffeic acid were significantly more abundant in species-rich grasslands compared to improved grasslands (12.76 ± 0.18 versus 11.54 ± 0.22; *t* = −2.99, p = 0.004) but did not differ from the average abundance found in herbal leys (*t* = 1.22, p = 0.22). Herbal leys were significantly more abundant in three anthelmintic compounds than the other grassland types: geraniol (9.66 ± 0.11; *t* = 3.14, p = 0.003), mannitol (13.63 ± 0.30; *t* = 6.91, p = 0.002), and chicoric acid (11.88 ± 0.41; *t* = 5.012, p < 0.001). The only antimicrobial compound significantly more concentrated in samples from improved grasslands than the other grassland types was trans-ferulic acid (12.57 ± 0.06; *t* = 3.98, p < 0.001). There was no significant difference in the following four antimicrobial compounds among grassland types: salicylic acid (*F*_2,40_ = 0.23, p = 0.80), carvacrol (*F*_2,40_ = 0.38, p = 0.69), coumarin (*F*_2,40_ = 0.69, p = 0.51), and homovanilic acid (*F*_2,40_ = 0.43, p = 0.65).

## Discussion

Untargeted metabolite profiling of 17 plant species showed that the grasslands studied were a rich source of antimicrobial and anthelmintic compounds. Our results highlight the key role of phylogeny in determining the metabolomic profiles across multiple plant species. This can be used as a guide to identify future plants and compounds that can be targeted for metabolic engineering to increase the production of antimicrobial/anthelmintic compounds. Analysis of 45 multi-species forage samples also confirmed for the first time that manipulating species-composition could be an effective method of increasing the abundance of known medicinal compounds in livestock feed.

### Cross-species patterns in the production of bioactive compounds

Plants closely related to one another at the family and order levels showed strong similarities in metabolomic profiles. Although there are currently no large-scale studies confirming that plants closely related may share similar metabolomic profiles, we do know that certain compounds are associated with specific orders or families (e.g. betalains in the order Caryophyllales, glycoalkaloids in the Solanaceae family)^31,32^. This may explain why some antimicrobial and anthelmintic compounds were generally abundant while others were species-specific. For example, hispidulin, mannitol, and rutin were found across all the plants sampled. These compounds may be genetically conserved over time (due to high bioactivity) and/or may have pathways that are grouped together. In contrast, thymol, lauric acid, and hydrocinnamic acid were much rarer and one compound, agropyrene, was only found in *Leucanthemum vulgare*. The latter is a major component of the essential oils produced by other plants from the Asteraceae family, such as tarragon (*Artemesia dracunculus* L.)^33^. Less ubiquitous compounds may reflect species-specificity which developed later (evolutionarily) in response to specific stresses and/or random genetic variation and selection^31,34^. In this study it was also found that bioactive compounds were the pre-cursors, intermediaries and end-products of multiple pathways. Many compounds appeared to be co-regulated, a feature also seen in *Arabidopsis* and fungi, which may suggest that attempts to increase the production of one target compound will require knocking out multiple genes and/or proteins^35,36^.

### Plants and compounds of biomedical interest

The analysis of the results indicated three wild plants and two cultivated legumes could be developed further as livestock feeds or as ‘factories’ for the production of specific compounds of biomedical interest. *F. ulmaria*, *C. nigra*, and *P. vulgaris* consistently contained high levels of multiple antimicrobial compounds’. *F. ulmaria* in particular contained the highest number of novel compounds and was metabolically the most dissimilar from the other plants we sampled. These plants also produced three compounds which could be purified and developed further as anti-biofilm agents: rosmarinic acid, carvacrol, and thymol. These compounds have been shown to disrupt quorum sensing and biofilm matrices^37,38^. Carvacrol and thymol are the most bioactive of all the compounds we identified and have Minimum Inhibitory Concentration (MIC) values of 0.04–0.32 μg/mL^39,40^. Our analyses also identified potential new compound targets in *M. sativa* and *O. viciifolia*. Current research on both crops has focused on increasing tannin content as a form of biocontrol for intestinal parasites^41^. We found these plants also contain other compounds known to decrease parasitic infection by decreasing mobility and egg survival (saponins, caffeic acid, thymol, geraniol)^42–44^. Increasing the production of these compounds could also decrease parasitic burdens in livestock without the ill effects tannins can have on livestock productivity (e.g. weight loss). A large proportion of compounds in the plants analyzed in this study remain unknown and may yet provide a new source of pharmaceuticals and feed additives for the future. Investigation of this potential was beyond the scope of this study.

### Sward manipulation has significant landscape-level effects

Our results show that manipulating the species-composition of grasslands at the landscape level is likely to have a considerable effect on the bioactive compounds found in mixed swards. The high concentration of antimicrobial compounds in species-rich grasslands is likely a reflection of their high vegetation diversity. These grasslands contain on average 15–40 (or more) plant species. In comparison, modern agriculturally ‘improved’ grasslands contain 1–5 species at most. The one antimicrobial compound strongly associated with improved grasslands was trans-ferulic acid. Trans-ferulic acid is involved in lignin biosynthesis; increased levels of this compound may be due to the abundance of grasses (e.g. *L. perenne*) that characterize improved grasslands^45^. Adding specific plants with particular compounds of interest may also an effective way to boost the nutraceutical value of forage. The herbal leys we analyzed contained the highest levels of multiple anthelmintic compounds. They were specifically planted with cultivated forage crops thought to have a high-tannin content (*M. sativa*, *O. viciifolia*, and *C. intybus*)^46–48^. The results suggest commercial meadow and pasture seed mixtures can be optimized to improve livestock health. All 17 plants screened in this study are found in grasslands and consumed by livestock without ill effects. The secondary metabolites in grassland plants target multiple aspects of microbial and parasitic metabolism and reproduction. The synergy of these compounds may prove a more effective, long-term defense against microbial and parasitic pathogens compared to current pharmaceuticals.

## Conclusion

Microbial and parasitic pathogens resistant to synthetic drugs are a threat to agriculture, food security and global health yet phytochemicals may prove a powerful weapon against their spread. We found that grassland plants are a rich source of antimicrobial and anthelmintic compounds and a very rich source of uncharacterized chemicals that may contain serve as the source of new medicines. Metabolomic profiles across species were largely dictated by phylogeny. Specific wild plants, namely *F. ulmaria*, *C. nigra*, and *P. vulgaris*, contained elevated levels of known antimicrobial compounds. Such plants could be included into livestock feed or specific compounds from these plants could be introduced into current feeds through metabolic engineering. Our research provides the only evidence to date that manipulating sward composition can have an effect on the overall availability of antimicrobial and anthelmintic compounds in forage. Future research should focus on how to exploit the rich source of natural compounds found in native grasslands in order to reduce reliance on antimicrobial drug use in animal and human populations.

### Data availability

All data used in this manuscript can be found in the Supplementary Data files.

## Electronic supplementary material

Supplementary Information

Supplementary Dataset 1

Supplementary Dataset 2
