# Supplementary material for: Targeted and Untargeted Metabolic Profiling of Wild Grassland Plants identifies Antibiotic and Anthelmintic Compounds Targeting Pathogen Physiology, Metabolism and Reproduction
Source: Sci Rep. 2018 Jan 26;8:1695. doi: 10.1038/s41598-018-20091-z (PMC5786025; doi:10.1038/s41598-018-20091-z)
Supplement: Supplementary file 1 — Supplementary Information [file 41598_2018_20091_MOESM1_ESM.pdf]

Targeted and Untargeted Metabolic Profiling of Wild Grassland Plants identifies Antibiotic and Anthelmintic Compounds Targeting Pathogen Physiology, Metabolism and Reproduction

Katherine E. French,[1*] Joe Harvey,[2] and James S.O. McCullagh[2*]

[1] Department of Plant Sciences, South Parks Road, University of Oxford, Oxford OX1 3RB, UK
[2] Department of Chemistry, Mansfield Road, University of Oxford, Oxford OX1 3TA, UK

Contact details: Email: katherine.french@plants.ox.ac.uk Tel: +44 07591089370

Contact details: James.mccullagh@chem.ox.ac.uk

Supplementary Materials

Table 1 Limits of detection (LOD) and limits of quantification (LOQ) for compounds identified in single or mixed plant samples with antimicrobial and/or anthelmintic properties.

**Limits of Detection and quantification**			
Compound	LOQ (ng/ml)	LOD (ng/ml)	R^2
Apigenin 7-O-neohesperidoside	8	2.7	0.995
Apigenin-7-O-glucuronide	8	2.7	0.994
Benzoic acid	200	66.7	0.999
Caffeic acid	8	2.7	0.999
Catechin hydrate	40	13.3	0.999
Chicoric acid	200	66.7	0.997
Chlorogenic acid	8	2.7	0.999
Epigallocatechin gallate	200	66.7	0.998
Fisetin	8	2.7	0.999
Gallic acid	40	13.3	0.999
Genistein	1.6	0.5	0.999
Genistin	40	13.3	0.999
Gentisic acid	1.6	0.5	0.999
Hesperetin	1.6	0.5	0.999
Hispidulin	8	2.7	0.999
Hydrocinnamic acid	8	2.7	0.999
Luteolin	1.6	0.5	0.999
Luteolin-7-O-glucuronide	40	13.3	0.994
Naringenin	1.6	0.5	0.999
P-Coumaric acid	40	13.3	0.997
Quercetin	0.0128	0.004	0.978
Quinic acid	0.064	0.02	0.999
Rhamnetin	8	2.7	0.999
Rosmarinic acid	8	2.7	0.999
Rutin hydrate	1.6	0.5	0.999
Salicylic acid	40	13.3	0.996
Trans-Ferulic acid	8	0.3	0.999

Table 2 Analytical precision found using eight replicates of a Quercetin standard.

**Precision**	
Replicate	Peak area
1	1088734877
2	1513126643
3	1067463481
4	1323959367
5	1439017757
6	1457244393
7	1107607722

8	1193506930
Relative standard Deviation	14.2

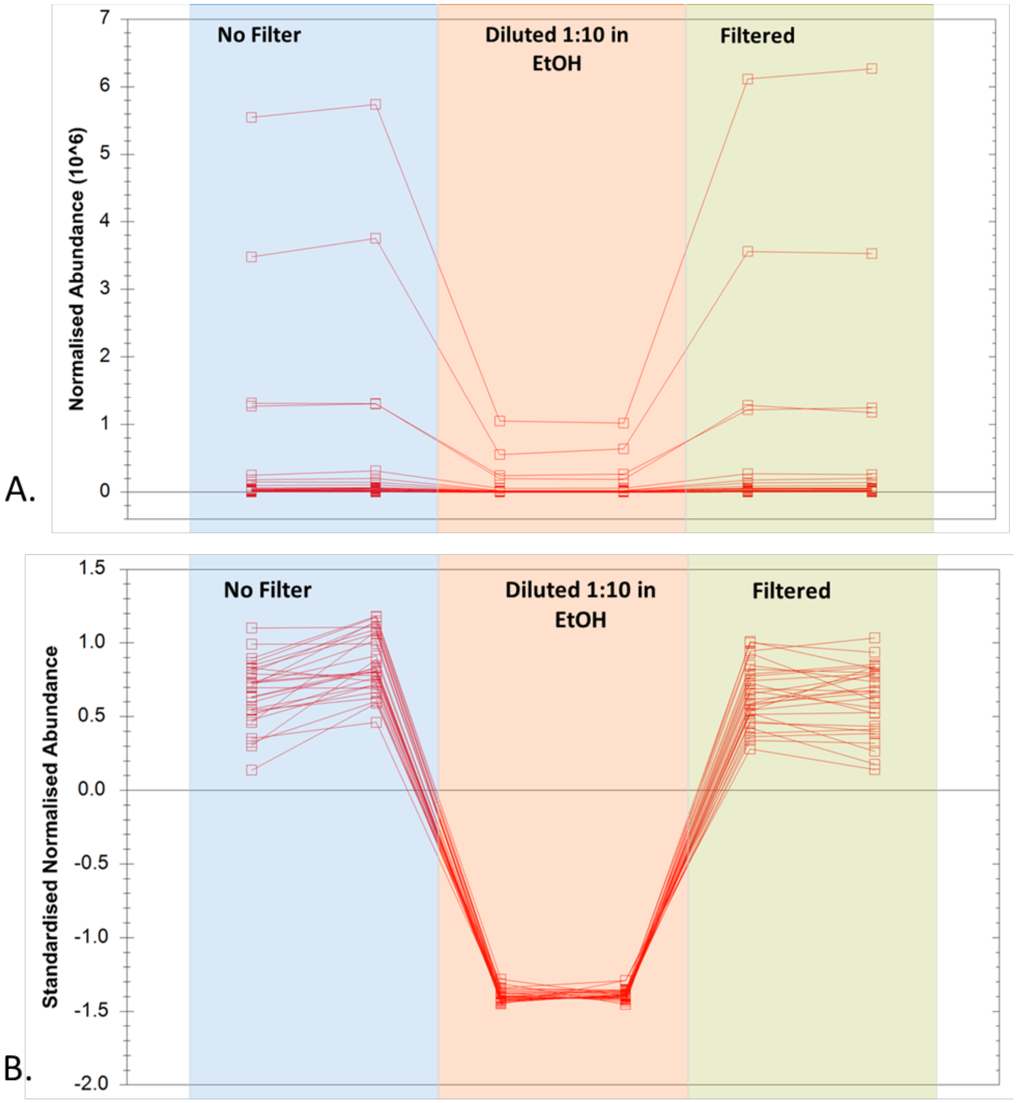

Figure 1 Effect of filtering on compound abundances. A. Normalized abundances of samples of samples without filtering, diluted 1:10 with 80% EtOH, and after filtering with a 0.45 um Millipore filter. B. Standardized normalized abundances of samples of samples without filtering, diluted 1:10 with 80% EtOH, and after filtering with a 0.45 um Millipore filter.

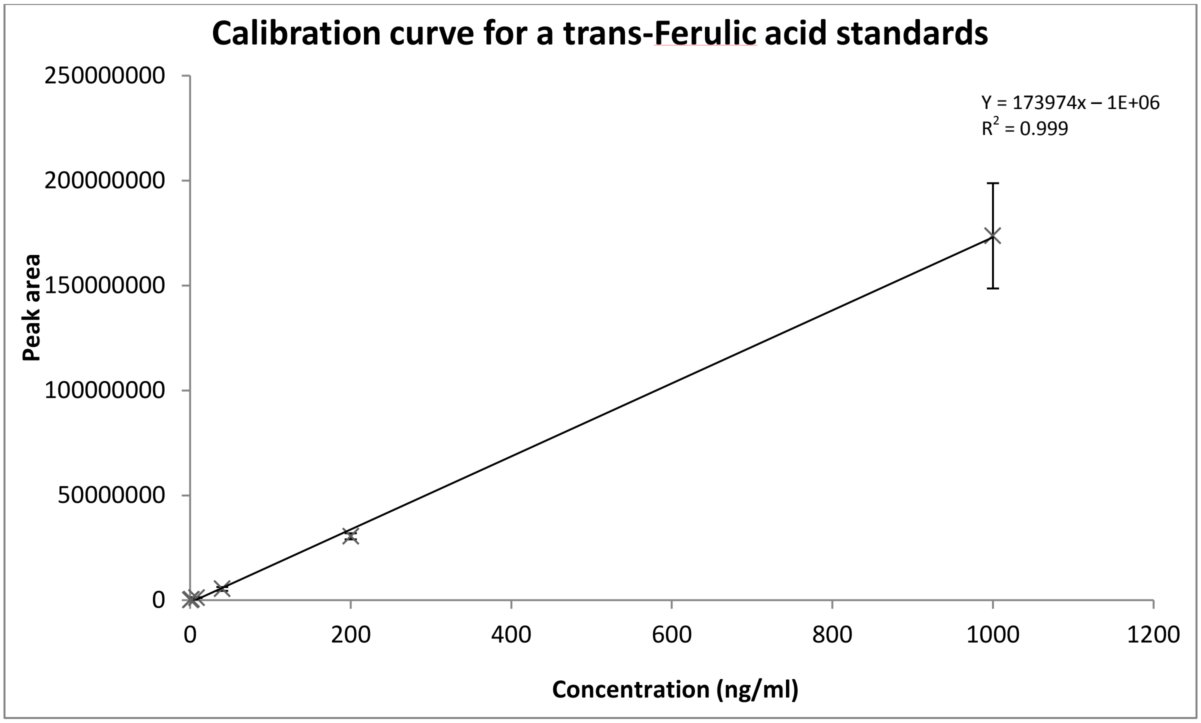

Figure 2 Calibration curve for trans-Ferulic acid authentic standard.

Table 3 Authentic standards and their database analytical properties used to identify compounds in plant extracts.

Compound ID	Neutral Mass	Adduct	Retention time (min)	Formula
**2,4,6-Tris(2-pyridyl)-s-triazine**	312.1123444	M+H	7.56	C18H12N6
**Baicalein**	270.0528233	M-H	8.65	C15H10O5
**Biochanin A**	284.067925	M-H	9.65	C16H12O5
**Capsaicin**	305.1990936	M-H	9.81	C18H27NO3
**Catechin**	290.079038	M-H	4.43	C15H14O6
**Daidzen**	254.0579087	M-H	7.30	C15H10O4
**Fisetin**	286.0477379	M-H	7.01	C15H10O6
**Galangin**	270.0528233	M-H	9.98	C15H10O5
**Gallic acid**	170.0215232	M-H	1.49	C7H6O5
**Hesperetin**	302.079038	M-H	7.90	C16H14O6
**Homovanilic acid**	182.0579087	M-H	4.93	C9H10O4
**Luteolin**	286.0477379	M-H	7.90	C15H10O6
**Luteolin-7-O-β-D-Glucuronide**	462.0798257	M-H	6.45	C21H18O12
**Myricetin**	318.0375671	M-H	6.91	C15H10O8
**Naringenin**	272.0684734	M-H	7.67	C15H12O5
**P-Coumaric acid**	164.047344	M-H	5.61	C9H8O3
**Protocatechuic acid**	154.0266086	M-H	2.80	C7H6O4
**Pyrogallol**	126.031694	M-H	1.46	C6H6O3
**Quercetin**	302.0426525	M-H	7.66	C15H10O7

**Quinic acid**	192.063388	M-H	0.77	C7H12O6
**Rutin hydrate**	610.1533845	M-H	6.47	C27H30O16
**Salicylic acid**	138.031694	M-H	6.18	C7H6O3
**Sinapic acid**	224.0684734	M-H	5.96	C11H12O5
**Syringic acid**	198.0528233	M-H	5.15	C9H10O5
**Tetracycline**	444.1532655	M-H	5.09	C22H24N2O8
**Vanilin**	152.047344	M-H	5.20	C8H8O3
**Apigenin**	270.0528233	M-H	8.48	C15H10O5
**Chrysin**	254.0579087	M-H	9.69	C15H10O4
**Hispidulin**	300.063388	M-H	8.54	C16H12O6
**Kaempferol**	286.0477379	M-H	8.37	C15H10O6
**T-Ferulic acid**	194.0573603	M-H	5.88	C10H10O4
**Vitexin**	432.1056466	M-H	6.17	C21H20O10
**Caffeic acid**	180.0422586	M-H	4.92	C9H8O4
**Genistin**	432.1056466	M+H	6.19	C21H20O10
**Gentisic acid**	154.0266086	M-H	3.85	C7H6O4
**Hydroxytyrosol**	154.0635425	M-H	2.90	C8H10O3
**Lauric acid / dodecanoic acid**	200.1781783	M-H	12.55	C12H24O2
**Malvidin chloride**	366.0511788	M+H	7.48	C17H15O7
**Arachidic acid**	312.3033788	M-H	15.63	C20H40O2
**Berberine chloride**	371.092984	M-H	4.70	C20H18NO4Cl
**Caffeine**	194.0809239	M-H	10.15	C8H10N4O2
**Chiocoric acid**	474.0798257	M-H	5.71	C22H18O12
**Chlorogenic acid**	354.0950819	M-H	4.70	C16H18O9
**Ellagic acid**	302.006267	M-H	6.67	C14H6O8
**Ellaidic acid**	282.2564286	M-H	14.60	C18H34O2
**Epigallocatechin gallate**	458.0849111	M-H	5.07	C22H18O11
**Ferulic acid**	194.0584571	M-H	5.88	C10H10O4
**Formononetin**	268.0730104	M-H	8.81	C16H12O4
**Emodin**	270.0533717	M-H	8.66	C15H10O5
**Homovanillyl alcohol**	168.0791926	M-H	13.58	C9H12O3
**O-Toluic Acid**	136.051881	M-H	5.84	C8H8O2
**Anabasine**	162.11515	M+H	16.81	C10H14N2
**Apigenin-7-O-glucuronide**	446.0843627	M-H	6.85	C21H18O11
**Harmine**	212.0944146	M-H	5.84	C13H12N2O
**Quinidine**	648.3670074	M+H	5.00	C40H48N4O4
**Quinine**	324.1837779	M+H	5.00	C20H24N2O2
**Tetrandrine**	622.3037385	M+H	5.76	C38H42N2O6
**Theophylline**	180.064177	M+H	4.10	C7H8N4O2
**Castanospermine**	189.0995594	M-H	0.72	C8H15NO4
**Catharanthine**	336.1832295	M+H	6.10	C21H24N2O2
**Cyclopamine**	411.313181	M+H	7.78	C27H41NO2
**Pilocarpine**	208.1206293	M+H	0.84	C11H16N2O2
**Theobromine**	180.064177	M-H	3.20	C7H8N4O2
**Tomatidine**	415.3444811	M+H	9.25	C27H45NO2
**Aconitine**	645.3143626	M+H	7.27	C34H47NO11
**Cocaine**	303.1465096	M+H	5.39	C17H21NO4

**Codeine**	299.151595	M+H	3.64	C18H21NO3
**Galanthamine**	287.151595	M+H	0.84	C17H21NO3
**Mescaline hcl**	211.1202949	M+H	4.29	C11H17NO3
**Morphine**	285.135945	M+H	0.80	C17H19NO3
**Tryptamine**	160.0995	M+H	3.64	C10H12N2
**Tyramine**	137.0835155	M+H	0.80	C8H11NO
**Ajmalicine**	352.1781441	M-H	6.14	C21H24N2O3
**Carvacrol**	150.1039166	M-H	9.41	C10H14O
**Cinchonidine**	294.1726649	M+H	4.92	C19H22N2O
**Conessine**	356.3186008	M-H	4.87	C24H40N2
**Geraniol**	154.1352167	M-H	8.11	C10H18O
**Tubocurarine)**	610.3042869	M-H	4.57	C37H42N2O6
**Benzoic acid**	122.0367794	M-H	6.36	C7H6O2
**Genistein**	270.0528233	M-H	8.08	C15H10O5
**Hydrocinnamic acid**	150.0680795	M-H	7.20	C9H10O2
**Isorhamnetin**	316.0583026	M-H	9.25	C16H12O7
**Pelargonidin**	271.0606483	M+H	8.11	C15H11O5
**Rhamnetin**	316.0583026	M-H	9.25	C16H12O7
**Rosmarinic acid**	360.0845173	M-H	5.78	C18H16O8
**Trans-Aconitic acid**	174.0164378	M-H	0.89	C6H6O6
**Amentoflavone**	538.0899965	M-H	9.85	C30H18O10
**Apigenin-7-O-neohesperidoside**	578.1635553	M-H	6.97	C27H30O14
**Cyanidin chloride**	322.0238672	M+H	8.00	C15H11O6
**Eriodictyol**	288.063388	M-H	7.15	C15H12O6
**Eriodictyol-7-O-glucoside**	450.1162113	M+H	6.03	C21H22O11
**Isoorientin**	448.1000128	M+H	5.99	C21H20O11
**Orientin**	448.1000128	M-H	5.99	C21H20O11
**Quercetin-3-O-glucoside**	464.0954758	M-H	6.63	C21H20O12
**Urolithin A**	228.0423	M-H	7.18	C13H8O4
**Urolithin B**	212.047344	M-H	8.15	C13H8O3
**Urolithin C**	244.0371732	M-H	6.59	C13H8O5

Table 4 Antimicrobial and anthelmintic compounds found in grassland plants. The table shows whether the compound has antimicrobial and/or anthelmintic properties and whether the biosynthetic pathway of the compound is known (designated by 'x').

Compound	Antimicrobial	Anthelmintic	Pathway	References
**Agropyrene**	x			Williamson and Evans, 1989
**Apigenin-7-O-glucuronide**	x		x	Duke, 1992; Newall et al., 1996; Recio et al., 1989
**Apigenin-7-O-neohesperidoside**	x		x	Duke, 1992; Newall et al., 1996; Recio et al., 1989
**Benzoic acid**	x		x	Duke 1992; Park 2001
**Biochanin A**	x		x	Flesar et al. 2009; Sklenickova et al. 2010
**Caffeic acid**		x	x	Cowan, 1999
**Carvacrol**	x	x		Baxter et al., 1998; Duke, 1992; Lagouri et al., 1993; Leung and Foster, 2003;

				Russell, 1986
**Catechin hydrate**		x		Cowan, 1999
**Chicoric acid**		x		Street et al. 2013
**Chlorogenic acid**	x		x	Lou et al. 2011; Kabir et al. 2014
**Chrysin**	x		x	Duke 1992
**Coumarin**	x		x	Baxter et al., 1998; Buchbauer et al., 1996; Newall et al., 1996
**Epigallocatechin gallate**		x	x	Cowan, 1999
**Fisetin**	x			Duke 1992
**Gallic acid**	x		x	Cowan, 1999
**Genistein**	x		x	Duke 1992
**Genistin**	x		x	Duke 1992
**Gentisic acid**	x		x	Duke 1992
**Geraniol**	x	x	x	Duke, 1992; Fleisher and Fleisher, 1991; Madaus, 1976; Muroi and Kubo, 1993
**Hesperetin**	x		x	Duke 1992
**Hispidulin**	x			Duke 1992
**Hydrocinnamic acid**	x		x	Duke 1992
**Luteolin**	x		x	Duke 1992; Joung et al. 2016
**Luteolin-7-O-D-glucuronide**	x		x	Duke 1992
**Malvidin chloride**	x			Duke 1992
**Mannitol**		x	x	Duke, 1992
**Myricetin**	x		x	Duke 1992
**Naringenin**	x		x	Duke 1992
**p-Coumaric acid**	x		x	Duke 1992
**Quercetin**	x		x	Baxter et al., 1998; Duke, 1992; Newall et al., 1996
**Quinic acid**	x		x	Duke 1992
**Rhamnetin**	x		x	Duke 1992
**Rosmarinic acid**	x		x	Duke 1992
**Rutin hydrate**	x			Duke 1992
**Salicylic acid**	x	x	x	Cowan, 1999
**t-Ferulic acid**	x		x	Duke 1992
**Thymol**	x	x		Duke, 1992; Sezik et al., 1993; Williamson and Evans, 1989

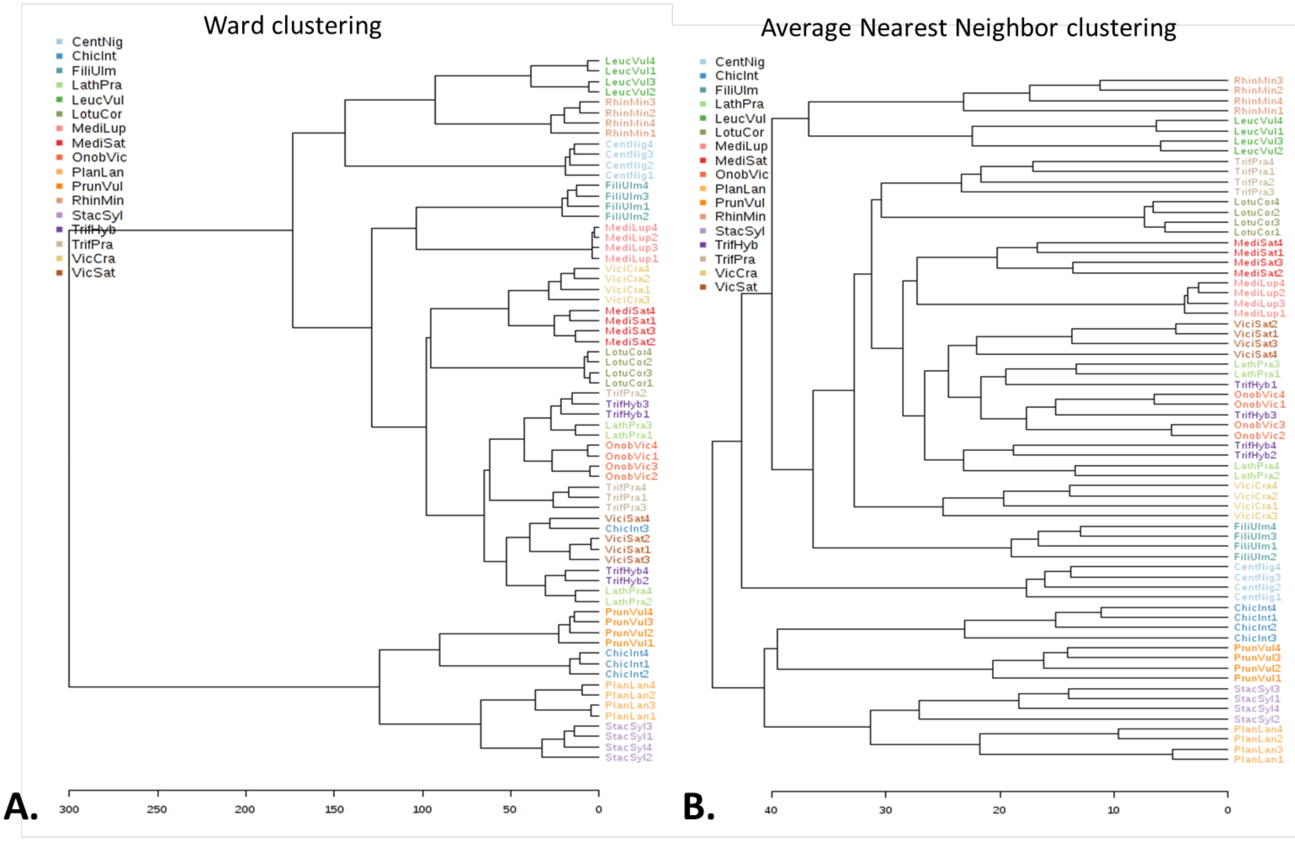

Figure 3 Chemotaxonomy of 17 grassland plants based on 51 compounds using Euclidean distances and (A) Ward clustering and (B) Average Nearest Neighbor.

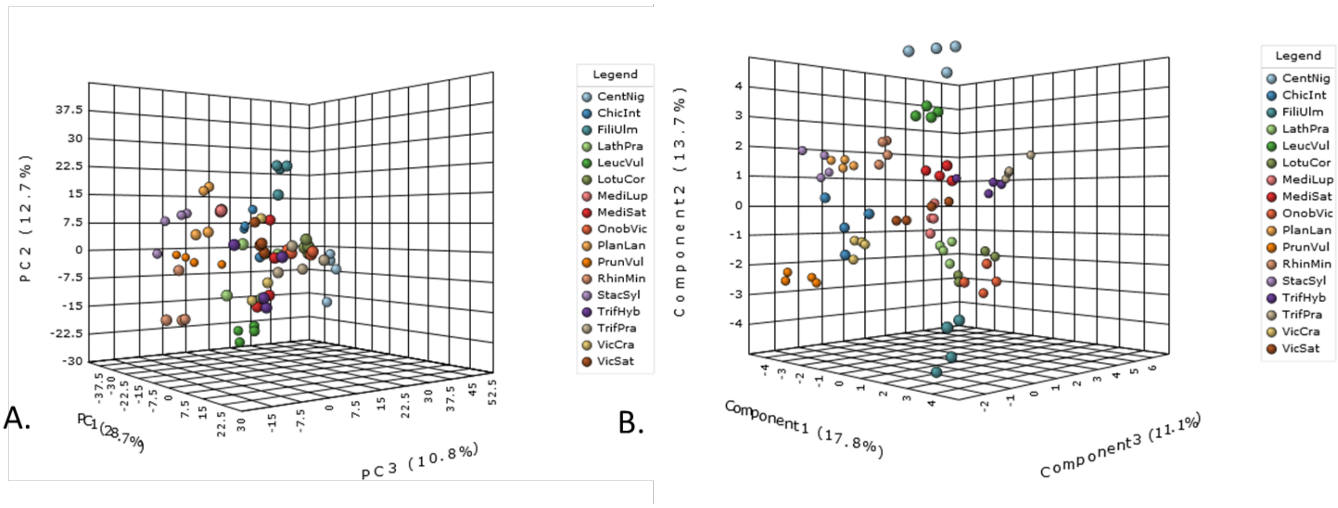

Figure 4 PCA (A.) and PLSDA (B.) showing the similarities and differences in metabolomic composition of 17 grassland plants. In all figures, samples from each plant are identified as the first four letters of the genus and first three letters of the species according to Linnean classification.

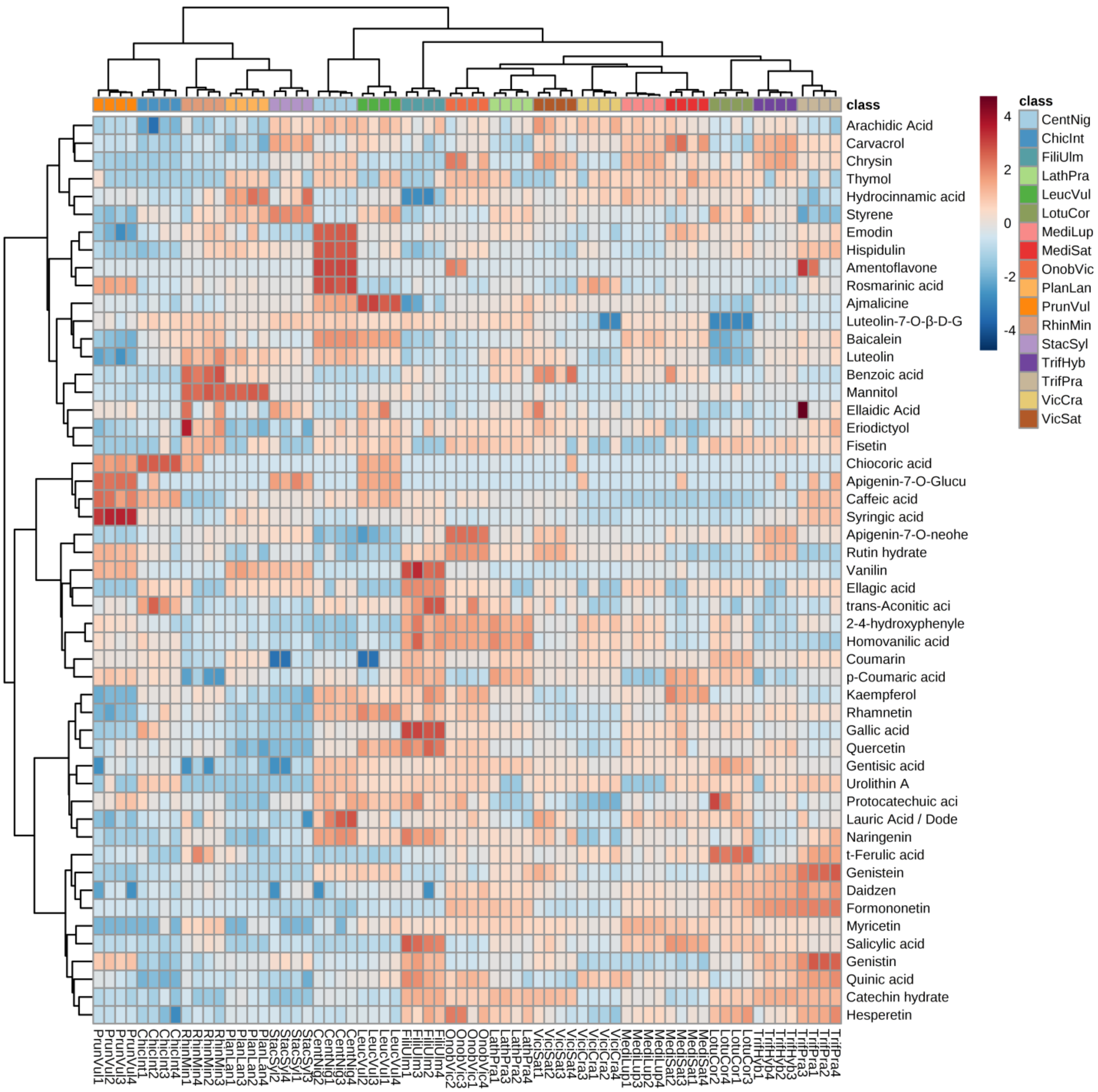

Figure 5 Heat map of 51 compounds found in 17 grassland plants based on Euclidean distances and Ward clustering. Samples are color coded by species labeled according to the first four letters of the genus and first three letters of the species.

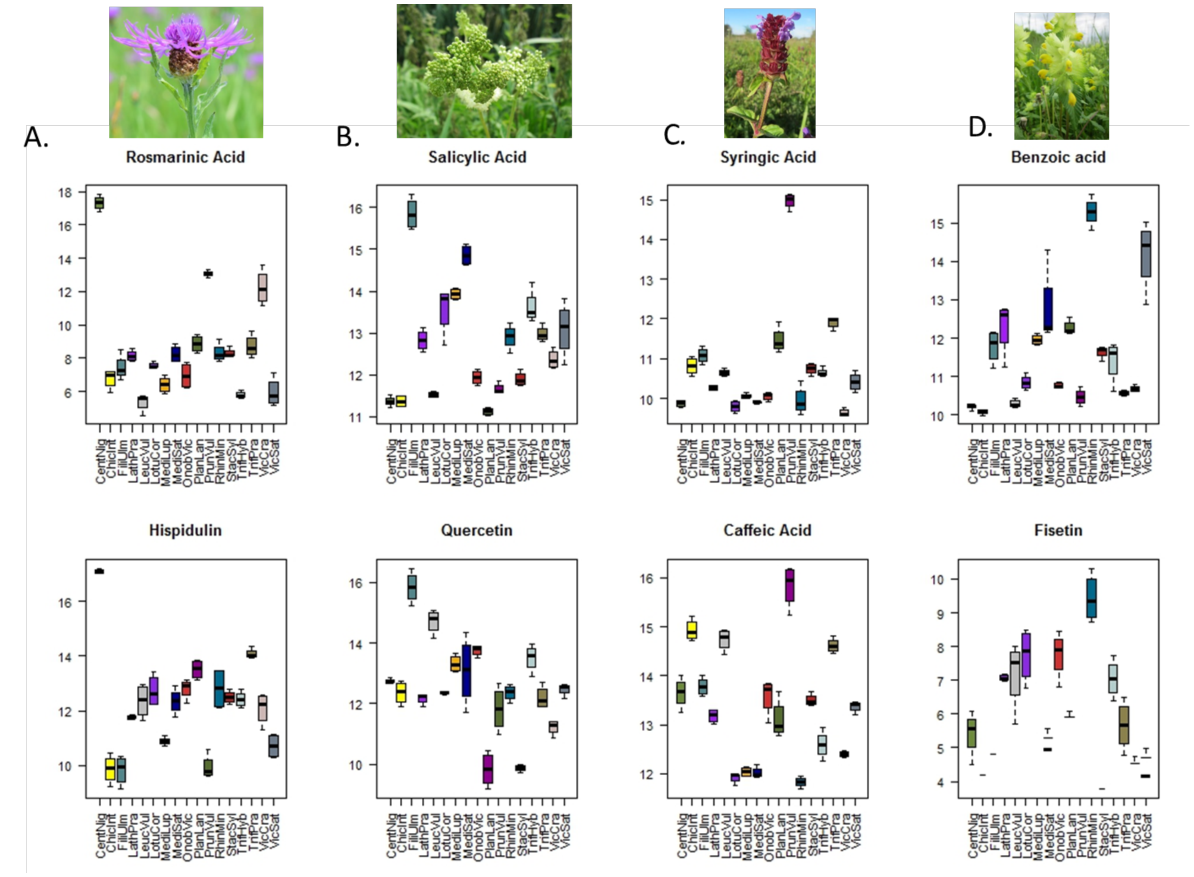

Figure 6 Concentration of antimicrobial and anthelmintic compounds in *Centaurea nigra* (A), *Filipendula ulmaria* (B), *Prunella vulgaris* (C) and *Rhinanthus minor* (D). Samples from each plant are identified as the first four letters of the genus and first three letters of the species according to Linnean classification. Compound concentrations are represented on a log scale.

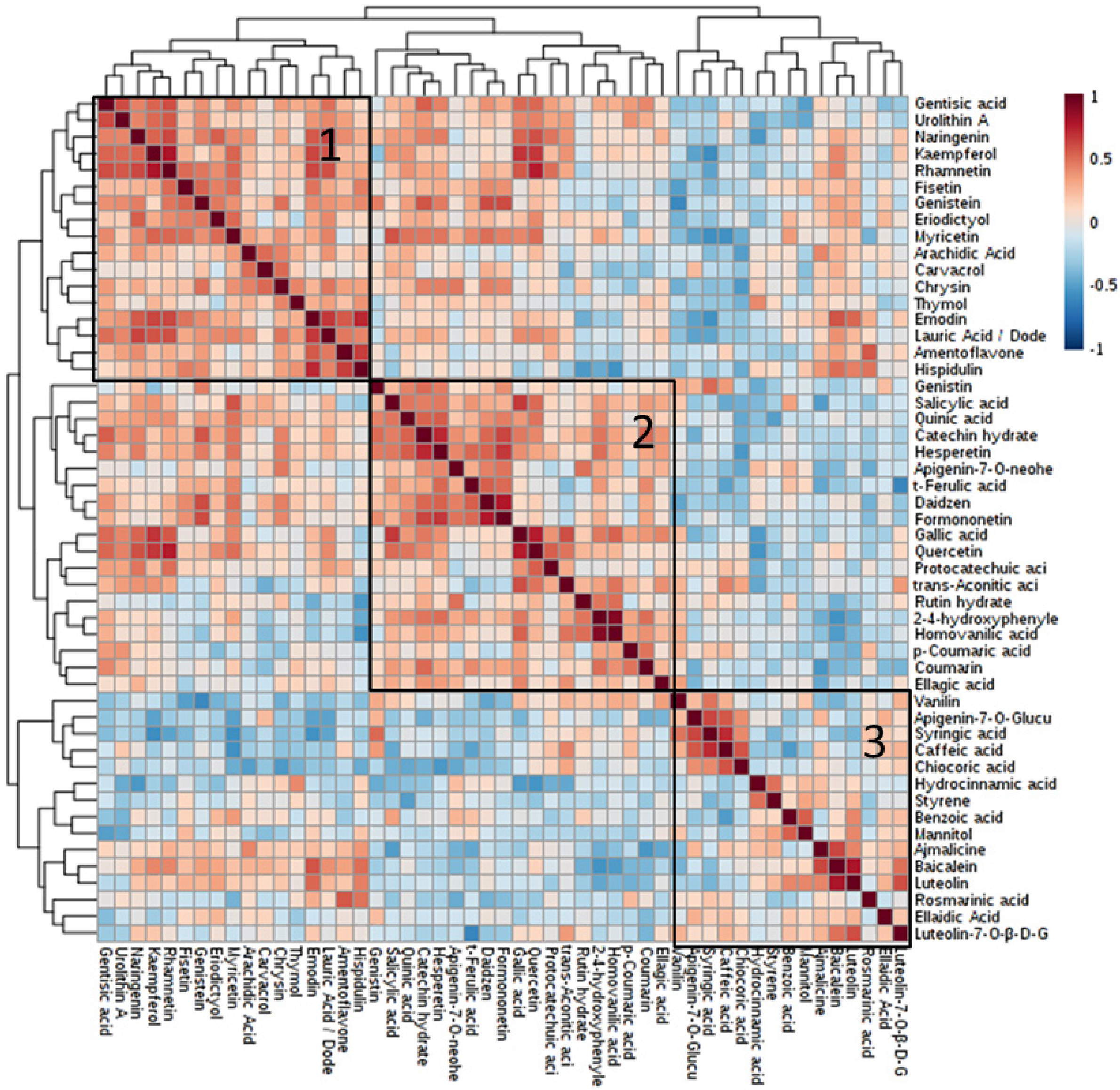

Figure 7 Heat map showing the correlations among compounds present in 17 grassland species. Pearson correlation (r) is used as the distance measure. Compounds fell into three main groups. The colors refer to the pair-wise correlation coefficient ranging from 1 (red) to -1 (blue).

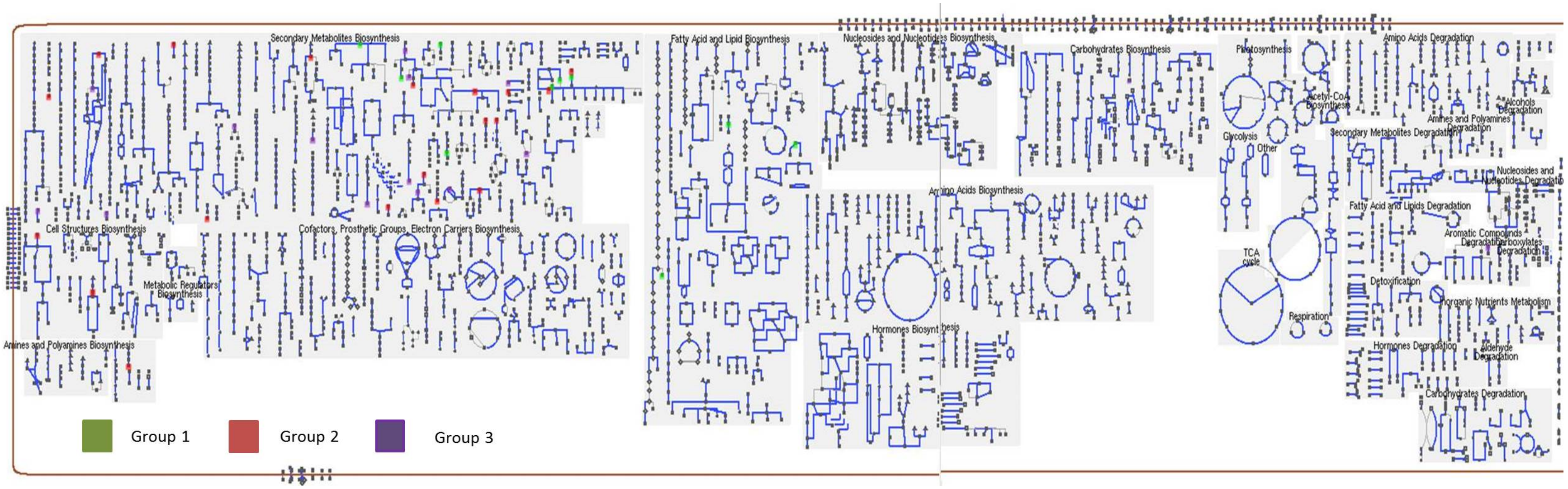

Figure 8 Metabolic map showing the three groups of highly correlated compounds and the pathways they belong to using *Arabidopsis* as a model. The map can be accessed at: http://pmn.plantcyc.org/overviewsWeb/celOv.shtml?zoomlevel=0&lon=1560&lat=29768&orgid=ARA&xnids=gentisic%20acid%0Aurolithin%20A%0Anarigenin%0Akaempferol%0Arhamnetin%0Afisetin%0Amyricetin%0Agenistein%0Aerodictol%0Aarachidic%20acid%0Ahispidulin%0Achrysin% .
Not all of the compounds have known pathways (e.g. thymol) and are not shown on the map.
